# SARS-associated Coronavirus Infection in Teenagers

**DOI:** 10.3201/eid1002.030485

**Published:** 2004-02

**Authors:** Gee-Gwo Yang, Shinn-Zont Lin, Kuang-Wen Liao, Jen-Jyh Lee, Lih-Shinn Wang

**Affiliations:** * Hualien Tzu-Chi Medical Center, Hualien, Taiwan

**To the Editor:** A global outbreak of severe acute respiratory syndrome (SARS) was reported in March 2003 (*1*). Most reported cases were in adults. Hong Kong, however, reported 10 pediatric cases (*2*) with less aggressive clinical courses.

The disease became endemic in Taiwan by the end of April 2003 (*3*). Hualien City, a geographically secluded city in eastern Taiwan, had nine pediatric cases, all mild. The cases occurred in Tzu-Chi High School, a private boarding school for 830 students 12 to 18 years of age, all of whom live in the same building and eat daily meals together in the school cafeteria. On April 28, when a student (case-patient 1) visited the school nurse on the first day that he had a fever, an infection specialist from affiliated Tzu-Chi Medical Center immediately responded. The specialist discovered that this student’s close friend in the same class (case-patient 2) was already febrile. Case-patient 2, a Hong Kong resident who leaves Taiwan for Hong Kong every 3 months, had visited Hong Kong twice in March and April 2003. Both students were isolated in the hospital on April 28.

Tzu-Chi Medical Center began a search for other febrile students. On April 29, seven more schoolmates were found to have fever >38°C. All were identified on their first day of becoming febrile and were immediately isolated in the hospital. All nine schoolmates underwent chest x-ray examinations and were tested for SARS-associated coronavirus (SARS-CoV) by reverse transcription–polymerase chain reaction (RT-PCR) (*4*) and DNA sequencing. The tested length for SARS-CoV was 340 bp in the RNA-dependent polymerase region. Those teenagers with diarrhea were tested for Norovirus in their stool by RT-PCR. For those teenagers with cough, throat swabs were cultured for influenza and parainfluenza virus.

To reduce the risk for false-positive PCR results, we followed measures to avoid contamination during specimen handling and processing. Two primer sets were used for RT-PCR according to Ksiazek (*4*) and Drosten (*5*). The targets are located in the RNA-dependent RNA polymerase gene at different regions, which are separated by approximately 3,000 bp. The laboratory used in RT-PCR analysis is not involved in viral culture or extraction preparation and is located far away from the laboratory for RNA extraction to avoid contamination.

Negative-control cDNA was included in each analysis and confirmed that no contamination had occurred. Two operators manipulated RT-PCR analysis for two specimens from the same sample. The specimens were analyzed in different rooms with independent reagents for assurance. Real-time RT-PCR instead of nested RT-PCR was used.

Six schoolmates were positive for SARS-CoV by RT-PCR, confirmed later by DNA sequencing for replicase. The tested DNA sequence was >99% identical with a published SARS-CoV sequence. Norovirus was identified in one teenager’s stool by RT-PCR; this virus belonged to genogroup I by testing partial cDNA sequence for capsid protein. The tested length was 555 bp, and the virus was 96% identical to strain KU4aGI. Culture of a throat swab for influenza and parainfluenza virus did not grow any virus.

The initial signs and symptoms of the nine teenagers were self-reported fever (9/9, range 37.8°C–39.4°C), cough (4/9), general malaise (4/9), diarrhea (4/9), rhinorrhea (3/9), headache (2/9), chills (2/9), sore throat (2/9), and myalgia (1/9). Cough was productive in three schoolmates and dry in one. Chest x-ray results were normal for eight teenagers but showed linear interstitial pneumonia for one teenager. Four schoolmates took ribavirin for <2 days. Only the teenager with pneumonia was treated with both ribavirin and clarithromycin, for 12 days. The other four schoolmates did not take medication. All nine schoolmates became afebrile by the third day. Seven schoolmates were completely asymptomatic in 3 days. Two other schoolmates showed improvement and had normal values of all repeated laboratory tests in 5 days; however, they still had mild coughs on the seventh day, when they were discharged. The one teenager with interstitial pneumonia also had a normal chest x-ray result on the fifth day. All nine teenagers were discharged after 1 week of hospitalization and were continuously isolated in a special dormitory for another 2 weeks. No new cases of fever have occurred in Tzu-Chi High School in the 2 months since these patients’ isolation.

Case-patient 2 was considered the index patient for SARS-CoV infection because of his travel history to Hong Kong. Six schoolmates with fever were confirmed by real-time RT-PCR and DNA sequences to have SARS-CoV infection. For students with diarrhea, only one case had coinfection with Norovirus. Influenza and parainfluenza viral infection was ruled out for students with cough. Because the nine ill schoolmates were isolated, no more cases of fever occurred in the school. All epidemiologic, molecular, and clinical studies showed evidence for SARS-CoV infection.

Worldwide, SARS-CoV infection has been clinically severe, characterized by respiratory distress and a 15% average mortality rate (*6*–*8*). Reported series of SARS with high mortality rates have involved mainly adults. Theoretically, subclinical or mild illness could be present and easily overlooked, and thus death rates could be overestimated.

The schoolmates in our series had mild illnesses and were identified only because of a special situation. On May 7, 2003, the World Health Organization (WHO) estimated that the case-fatality rate for SARS ranged from 0% to 50%, depending on the age group affected (*8*); for teenagers or younger children, the case-fatality ratio was <1%. Our teenagers with presumed SARS-CoV infection had very mild courses. This benign course was not related to treatment: only one teenager had a full course of ribavirin treatment, and most of the teenagers had either no specific medications or medications for <2 days. Our preliminary presumption for the benign course was the patients’ young ages. The benign course of SARS-CoV infection in our teenage students supports the WHO finding of less-severe disease in younger persons. These reasons should be explored more fully and may facilitate the development of more effective treatment and prevention programs in persons of all ages.
